# A Rare Case of Antinuclear Antibody (ANA)-Negative Lupus Nephritis

**DOI:** 10.7759/cureus.41480

**Published:** 2023-07-06

**Authors:** Nayaab Bakshi, Talha Munir, Michael Guma, Kara B Chenitz

**Affiliations:** 1 Internal Medicine, Saint Michael's Medical Center, Newark, USA; 2 Nephrology, East Orange Veterans Affairs Medical Center, East Orange, USA

**Keywords:** ana negative, anti-nuclear antibody, ana, lupus nephritis, systemic lupus erythematosis

## Abstract

Because most patients with lupus nephritis have a positive antinuclear antibody (ANA), ANA-negative lupus nephritis is a rare complication of systemic lupus erythematosus (SLE). In the 2019 European Alliance of Associations for Rheumatology/American College of Rheumatology (EULAR/ACR) classification criteria for SLE, a negative ANA precludes further work-up of SLE. The following case discusses a patient with multiple negative ANA titers but was diagnosed with SLE based on the findings of the kidney biopsy showing lupus nephritis. Though ANA was negative, anti-double-stranded DNA (anti-dsDNA) and anti-Sjogren's syndrome-A (anti-SS-A) antibodies were high. This case highlights the nuances of SLE and further illustrates the challenges in making a diagnosis of SLE when serology alone is relied on for screening.

## Introduction

Systemic lupus erythematosus (SLE) is an autoimmune condition that can affect multiple organ systems and understanding its manifestations is imperative. The incidence of SLE is 5.1 per 100,000 person-years and the prevalence is 161,000 people [1].

Patients with suspected lupus are screened with an antinuclear antibody (ANA) test. Because 98% of patients with SLE have a positive ANA, ANA is “the most sensitive diagnostic test for confirming the diagnosis of the disease” [2]. If the ANA is negative, it is highly unlikely that the patient has SLE. If ANA is positive, defined as the titer of 1:80 or greater on HEp-2 cells or equivalent, further investigation using clinical features and serology is performed to potentially diagnose lupus [3].

Amongst these criteria is lupus nephritis. Present in about two-thirds of patients, lupus nephritis is a serious complication of SLE [4]. African Americans, Latinos, and Asian Americans, especially women between 15 and 44, are more likely to get lupus nephritis [4-5]. Compared to patients with SLE without renal manifestations, patients with both SLE and lupus nephritis have increased mortality with 10% of patients progressing to end-stage renal disease [6].

Characteristics of lupus nephritis include hypertension, peripheral edema, cardiac decompensation, renal injury, hematuria, and proteinuria. Lupus nephritis is diagnosed by kidney biopsy [7]. The likelihood of lupus nephritis occurring in the setting of ANA being negative is important because ANA is used as an initial screening tool for autoimmune conditions like SLE. Therefore, a negative ANA may be grounds for ruling out SLE in a physician’s differential in a patient with nephritis. The following case illustrates a rare occurrence of ANA-negative lupus nephritis.

## Case presentation

A 27-year-old white woman with a history of undetermined anemia presented to the hospital with complaints of fatigue, malaise, pleuritic, sub-sternal chest pain, shortness of breath, and palpitations for three days. The patient denied any history of fevers, rashes, lower extremity swelling, ulcers, arthritis, or seizures. The remainder of the review of the systems was negative. She had no previous history of hypertension or renal disease and did not have any significant family history. The patient’s blood pressure on admission was 201/103 mmHg. Her temperature, heart rate, respiratory rate, and oxygen saturation were within normal limits. Additionally, the physical examination was unremarkable, notably negative for murmurs, rubs, gallops, oral ulcers, alopecia, or rash. The CT angiogram chest with contrast showed mild cardiomegaly with small pericardial effusion, bilateral axillary lymphadenopathy, and no pulmonary embolism.

Initial laboratory results showed normal blood urea nitrogen and creatinine, as well as normocytic anemia. Urinalysis revealed 4+ proteinuria, 2+ blood, 3-10 RBCs per high-powered field, hyaline casts, and >25 white blood cells (Table 1). The urine protein to creatinine ratio suggested 1.93 g of protein excreted in 24 h. The patient tested negative for Hepatitis B, C, and human immunodeficiency virus. An ANA test was ordered four times on separate days as part of the workup for her heavy subnephrotic range proteinuria, which resulted in negative four times.

**Table 1:** Laboratory results. mmol/L, millimoles/liter; mg/dL, milligrams/deciliter; U/L, units/liter; g/dL, grams/deciliter; mg/dL, milligrams/deciliter; fL, femtoliter; pg, picograms; uL, microliter; ug/dL, micrograms/deciliter; uL, microliter; HPF, high powered field

Laboratory test	Result	Laboratory test	Result	Urinalysis	Result
Sodium	136 mmol/L	Hemoglobin	7.8 g/dL	Bacteria	> 30 many/HPF
Potassium	3.3 mmol/L	Mean corpuscular volume	96.3 fL	Bilirubin	2+
Chloride	98 mmol/L	Mean corpuscular hemoglobin	30.9 pg	Blood	2+
Carbon dioxide	30 mmol/L	White blood cells	8000/uL	Casts	Hyaline
Anion gap	8 mmol/L	Red cell distribution width	15.9%	Glucose	Trace
BUN	12 mg/dL	Reticulocyte count	8.0%	Ketones	1+
Creatinine	0.6 mg/dL	Lactate dehydrogenase	208 U/L	Leukocyte esterase	Positive
AST	34 U/L	Iron	72 ug/dL	Protein	4+
ALT	35 U/L	Iron saturation	36.7%	Red Blood Cells	3-10 (few)/HPF
Alkaline phosphatase	112 U/L	Total iron binding capacity	196 ug/dL	White Blood Cells	>25 (many)/HPF
Total protein	8.5 g/dL	Ferritin	333.1 ng/mL		
Albumin	4.1 g/dL	Platelets	501,000/uL		
Total bilirubin	0.9 mg/dL				

**Figure 1:**
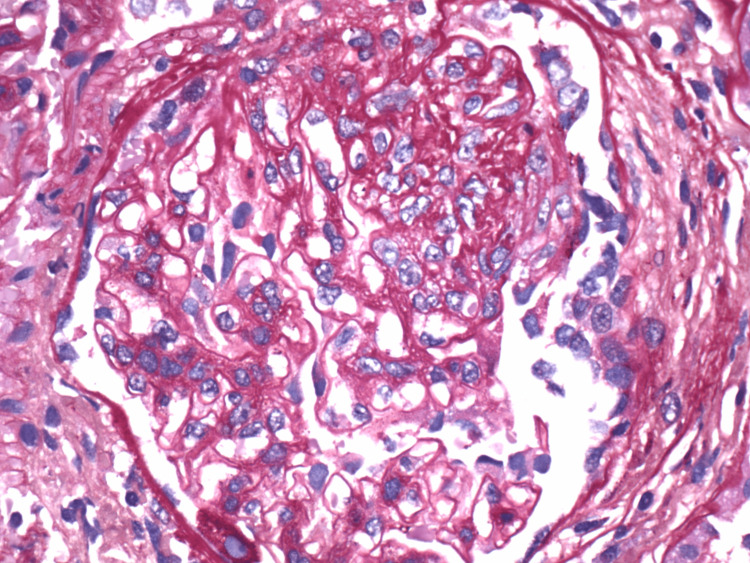
Renal biopsy pathology - fibrocellular crescent. Sections are stained with hematoxylin and eosin, periodic acid-Schiff, trichrome and Jones methenamine silver. Glomerulus showing a small fibrocellular crescent, and no clear-cut leukocyte infiltrates, hyaline deposits, fibrinoid necrosis, or thrombi were identified. There was minimal patchy interstitial inflammation affecting <5% of the cortical area. There was no significant tubular atrophy or interstitial fibrosis. Arteries display mild intimal fibrosis and no evidence of arteritis.

**Figure 2:**
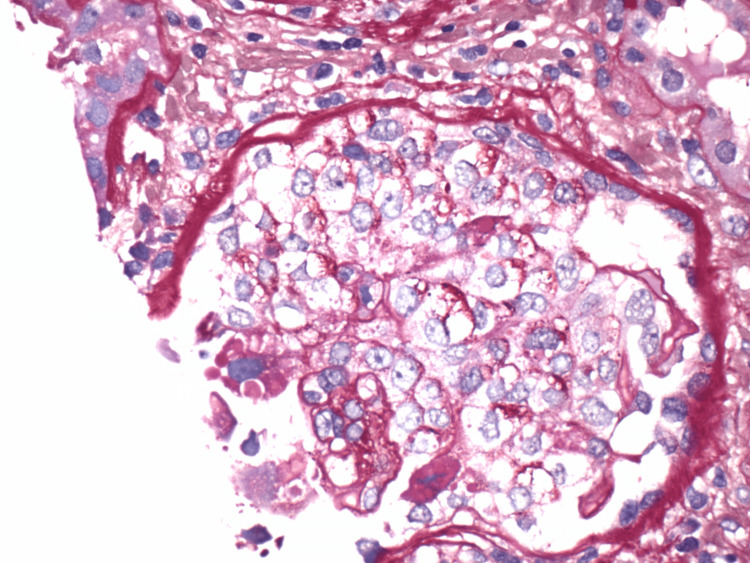
Renal biopsy pathology - crescent. One glomerulus showing a cellular crescent.

**Figure 3:**
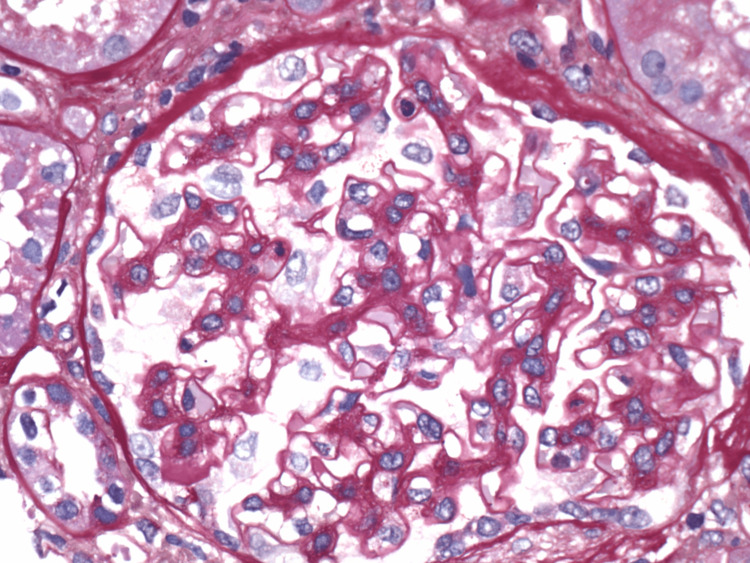
Renal biopsy pathology - diffuse mesangial hypercellularity. Glomeruli are normal and show mild diffuse mesangial hypercellularity.

**Figure 4:**
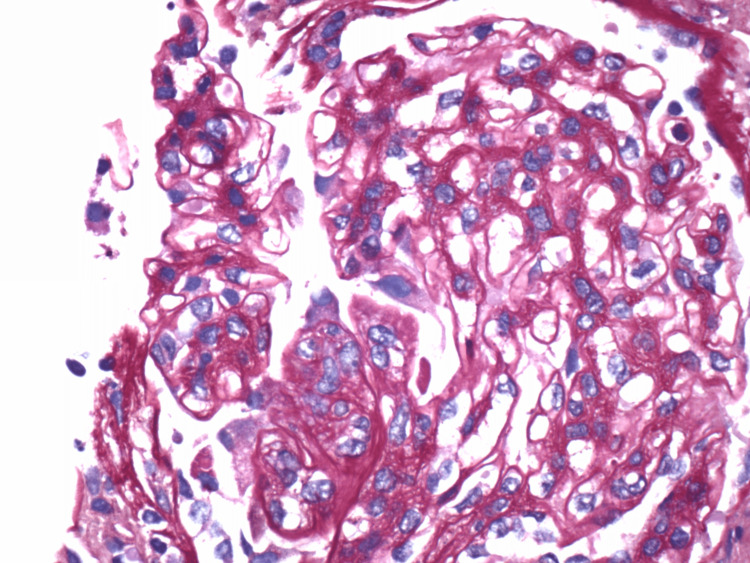
Renal biopsy pathology - segmental endocapillary hypercellularity.

**Figure 5:**
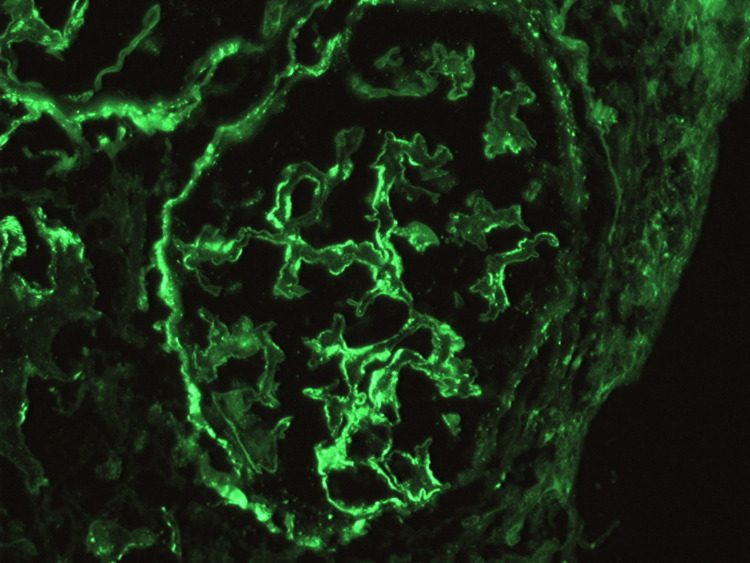
Renal biopsy pathology - immunofluorescence: IgG.

A kidney biopsy was performed, which showed focal endocapillary and extra capillary proliferative glomerulonephritis, consistent with lupus nephritis class III (with mild activity and no chronicity), minimal interstitial inflammation, and mild arteriosclerosis (Figures 1-5, Table 2). The ISN-RPS activity index was 4/24 and the chronicity index was 0/12. Sixteen glomeruli were seen, none of which showed segmental or global sclerosis. 

**Table 2:** Immunofluorescence results. IgG, immunoglobulin G; IgM, immunoglobulin M; IgA, immunoglobulin A; C3, C3 complement; gloms, glomeruli; seg, segment; gran, granular; mes, mesangial; GCW, glomerular capillary walls; TBM, tubular basement membrane; FBGN, fibrinogen; ALB, albumin

	Glomeruli	Tubules	Interstitium	Vessels
IgG	5 gloms 1-2+ seg gran mes & GCW	TBM 1+ gran	Negative	Negative
IgM	5 gloms 1+ seg gran mes & GCW	Negative	Negative	Negative
IgA	5 gloms +/- seg gran mes & GCW	Negative	Negative	Negative
C3	5 gloms 1+ seg gran mes & GCW	TBM’s 1+ gran	Negative	Arteriole walls +
C1	5 gloms +/- seg gran mes	Negative	Negative	Negative
FBGN	5 gloms negative	Negative	Negative	Negative
ALB	5 gloms negative	Negative	Negative	Negative
KAPPA	5 gloms 2+ seg gran mes & GCW	TBM’s 1+ gran	Negative	Negative
LAMDA	5 gloms 1+ seg gran mes & GCW	TBM’s +/- gran	Negative	Negative

After the kidney biopsy, an additional autoimmune workup was performed as shown in Table 3. Notably, the anti-double stranded DNA (anti-dsDNA) antibody and anti-SS-A (anti-Sjogren's syndrome-A) antibody were high and C3 and C4 were normal. Erythrocyte sedimentation rate and C-reactive protein were both high. The patient was diagnosed with ANA-negative lupus nephritis and she was discharged on lisinopril, mycophenolate, and prednisone.

**Table 3:** Autoimmune laboratory results. mg, milligram; mm/h, millimeter/h; dL, deciliter; AI, antibody index; GPI, glycoprotein I; IU/mL, international units per milliliter; IgA, immunoglobulin A; IgM, immunoglobulin M; IgG, immunoglobulin G; DNA, deoxyribonucleic acid; anti-SS-A, anti-Sjogren's syndrome-A; anti-SS-B, anti-Sjogren's syndrome-B; Anti-Scl-70, topoisomerase I; ANA, anti-nuclear antibody

Laboratory Test	Result	Reference range
ANA (by indirect immunofluorescence) (sent 4 times on separate days)	All 4 ANA tests were negative	
Anti-centromere B antibody	< 0.2 (negative)	0.0-0.9 AI
Anti-Smith antibody	< 20 (negative)	0.0-0.9 AI
Anti-Scl-70 antibody	< 0.2 (negative)	0.0-0.9 AI
Anticardiolipin antibody IgG	23 (low to medium positive)	Negative: < 15; Indeterminate: 15-20 ; low to medium positive: 21-80; High positive: > 80
Anticardiolipin antibody IgM	12 (negative)	Negative: < 13; Indeterminate: 13-20 ; low to medium positive: 21-80; High positive: > 80
Beta-2 glycoprotein I antibody IgG	< 9 (negative)	0-20 GPI IgG units
Beta-2 glycoprotein I antibody IgM	< 9 (negative)	0-32 GPI IgM units
Beta-2 glycoprotein I antibody gA	10 (negative)	0-25 GPI IgA units
Lupus Anticoagulant	Not detected	
Anti-citrullinated protein antibody IgG/IgA	23	Negative: < 20; weak positive 20-39; moderate positive 40-59; Strong positive: > 59
Rheumatoid factor	18.9 (high)	0 -15 IU/mL
Anti-double-stranded DNA antibody	71 (high)	Negative: < 5; equivocal: 5-9; positive: > 9
Anti-ribonucleoprotein (anti-RNP) antibody	0.3 (negative)	0.0-0.9 AI
Sjogren’s Anti-SS-A	> 8.0 (high)	0.0-0.9 AI
Sjogren’s Anti-SS-B	< 0.2 (negative)	0.0-0.9 AI
Antichromatin antibody	6.8 (positive)	0.0-0.9 AI
Anti-Jo-1 antibody	< 0.2 (negative)	0.0-0.9 AI
Anti-centromere B antibody	< 0.2 (negative)	0.0-0.9 AI
C3 complement	76 (normal)	75-175 mg/dL
C4 complement	16 (normal)	14-40 mg/dL
Erythrocyte sedimentation rate	> 145 (high)	0-20 mm/h
C-reactive protein	2.4 (high)	0-0.8 mg/dL

## Discussion

Among patients who have SLE, 1%-5% have a negative ANA, a test known to have a false-positivity rate of 15% [8]. ANA titers can vary, and ANA can be falsely positive, with 20%-30% of the population having a positive ANA, depending on the technique being used. Amongst techniques like enzyme immunoassay and multiplex immunoassay, ANA by indirect immunofluorescence, which gives a titer, a pattern, and tests antibodies for multiple cellular antigens, is the gold standard recommended by the American College of Rheumatology as it is the most sensitive test [9]. Given the sensitivity of ANA being 98% for SLE and the majority of patients with SLE having a positive ANA, a negative ANA effectively rules out SLE. 

If we objectively assess our patient for SLE to the 2019 EULAR/ACR (European Alliance of Associations for Rheumatology/American College of Rheumatology) classification algorithm, our patient would not have qualified for further workup of SLE as she had four negative ANA levels via indirect immunofluorescence [3]. However, if the ANA negative result were circumvented, our patient would have fulfilled the additive criteria for lupus as her kidney biopsy showed class III lupus nephritis, scoring a 10, which alone could have qualified her for the SLE diagnosis (Table 4). Anti-dsDNA antibodies, greater than 90% specific to SLE and implicated in the pathogenesis of lupus nephritis, were positive [10]. Anti-cardiolipin IgG antibodies were low to medium positive. She additionally had proteinuria and pericardial effusion. If ANA were positive, per the algorithm, this patient would have an obvious diagnosis of SLE with a score of 27.

**Table 4:** Additive criteria recommended to aid in the diagnosis of SLE in patients with a positive ANA as per the 2019 EULAR/ACR classification. 2019 EULAR/ACR Classification Criteria for Systemic Lupus Erythematosus (SLE) [3]. Score = relative weight of each criterion attributed to SLE as per the classification system referenced above; a total score of greater than or equal to 10 suggests that a diagnosis of SLE is likely. ANA, anti-nuclear antibody

Criteria	Score	Criteria	Score
Fever	2	Oral ulcers	2
Leukopenia	3	Subacute cutaneous or discoid lupus	4
Thrombocytopenia	4	Acute cutaneous lupus	6
Autoimmune hemolysis	4	Pleural or pericardial effusion	5
Delirium	2	Acute pericarditis	6
Psychosis	3	Joint involvement	6
Seizure	5	Proteinuria (>0.5 g/24 h)	4
Non-scarring alopecia	2	Renal biopsy class II and V lupus nephritis	8
Anti-cardiolipin antibody, Anti-beta2GP1 antibody, lupus anticoagulant	2	Renal biopsy class III or IV lupus nephritis	10
		Low C3 or C4 complements	3
		Low C3 and low C4 complements	4
Anti-Smith antibody or anti-double-stranded DNA antibody	6	If total score ≥ 10, SLE is likely	

Anti-dsDNA antibody levels, well-known to cause immune complexes implicated in the pathogenicity of lupus nephritis, were sent retroactively after the kidney biopsy results showed lupus nephritis [11]. In the kidney biopsy, the chronicity index of 0/12 and activity index of 4/24 showed that there was no fibrosis or scarring, suggesting that the nephritis may have recently developed, further raising the possibility that the patient may have just developed lupus nephritis before the ANA was high enough to detect SLE. Therefore, using solely ANA to rule out SLE and possibly delay the workup of lupus nephritis may prevent earlier diagnosis and treatment, both of which are known to lead to a better prognosis for the patient. 

## Conclusions

This is an important case as it highlights the possibility of having SLE and lupus nephritis without having a positive ANA. ANA is generally the first test ordered to screen for SLE. When negative, SLE is generally not further pursued. Though ANA was negative, the patient had other features of lupus and a kidney biopsy was necessary to diagnose lupus nephritis. Knowing exceptions like this case exist encourages a nuanced approach to diagnosing SLE. 
